# OTX1 exerts an oncogenic role and is negatively regulated by miR129-5p in laryngeal squamous cell carcinoma

**DOI:** 10.1186/s12885-020-07279-1

**Published:** 2020-08-24

**Authors:** Xiu-Ping Tu, Hao Li, Liang-Si Chen, Xiao-Ning Luo, Zhong-Ming Lu, Si-Yi Zhang, Shao-Hua Chen

**Affiliations:** 1 Department of Otorhinolaryngology, Guangdong General Hospital & Guangdong Academy of Medical Sciences, Guangzhou, 510080 People’s Republic of China; 2 Sun Yat-sen University Cancer Center, Guangzhou, 510060 People’s Republic of China; 3 State Key Laboratory of Oncology in South China, Guangzhou, 510060 People’s Republic of China; 4 Collaborative Innovation Center for Cancer Medicine, Guangzhou, 510060 People’s Republic of China

**Keywords:** Orthodenticle homeobox 1, Laryngeal squamous cell carcinoma, MiR-129-5p, Tumor progression, Prognosis

## Abstract

**Background:**

Orthodenticle homeobox 1 (OTX1) is a transcription factor that plays an important role in various human cancers. However, the function of OTX1 in laryngeal squamous cell carcinoma (LSCC) is largely unknown. We aimed to explore the roles of OTX1 in LSCC and its possible molecular mechanism.

**Methods:**

The expression levels of OTX1 were assessed in LSCC cell lines and tissue samples. We further examined the effect of OTX1 on LSCC progression. The upstream regulator of OTX1 was identified using a computer algorithm and confirmed experimentally.

**Results:**

OTX1 was highly expressed in 70.7% (70/99) of LSCC tissue samples. The OTX1 expression in LSCC was significantly correlated with lymph node metastasis. High OTX1 expression in patients with LSCC was correlated with poor prognosis. Knockdown of OTX1 inhibited proliferation, colony formation, migration and invasion in LSCC cells. Knockdown of OTX1 inhibited tumor growth in a xenograft mouse model. Mechanistically, OTX1 might act as a direct target of miR-129-5p. OTX1 enhanced tumorigenicity and tumor growth both in vitro and in vivo.

**Conclusions:**

Our findings support that OTX1 is an oncogene in LSCC tumorigenesis and progression. Furthermore, OTX1 is a direct target of miR-129-5p in LSCC cells. Taken together, OTX1 is a promising diagnostic and therapeutic marker for LSCC.

## Background

Laryngeal squamous cell carcinoma (LSCC) is one of the most common malignant cancers to occur in the head and neck region. In China, the estimated incidence of LSCC was 26,400 new cases, while 14,500 related deaths occurred in 2015 [1]. In the United States, the incidence and mortality rates were 13,360 and 3660 per year in 2017, respctively [2]. Human papilloma virus infection, smoking, alcohol consumption and fried foods consumption are considered high-risk factors for the occurrence and development of laryngeal cancer [3–5]. At present, surgical resection, chemotherapy, and radiotherapy are considered the standard treatments for patients with LSCC [6, 7]. Despite advances in diagnosis and treatment, the prognosis of LSCC remains poor. Therefore, further understanding of the molecular mechanisms underlying the carcinogenesis and development of LSCC is important to identify new diagnostic markers and therapeutic targets for patients with LSCC.

Orthodenticle homeobox 1 (OTX1) is clustered on human chromosome 2p13, which includes five exons, encodes a protein containing 354 amino acids, and is widely distributed in human tissues [8, 9]. OTX1 is a transcription factor that exhibits high-affinity binding to TAATCC/T elements on target genes and plays an essential role in the process of embryo development, such as brain corticogenesis, neuron differentiation, sense organ development, and mammary gland development [10–16]. With the continuous progression in studies, the role of OTX1 in the carcinogenesis and development of human cancer has drawn increasing attention. It has been reported that overexpression and tumor-promoting effects of OTX1 are observed in hepatocellular carcinoma, colorectal cancer, and breast cancer [17–19]. Meanwhile, the activation of OTX1 expression occurs in aggressive non-Hodgkin lymphoma [20]. These findings suggest that OTX1 might be a potential oncogene and anti-cancer therapeutic target.

Based on these findings, we sought to investigate the possible roles of OTX1 in the oncogenesis and progression of LSCC as well as related molecular mechanisms.

## Methods

### LSCC tissue specimens and patient follow up

In this study, a total of 99 LSCC tissues were obtained from patients who underwent surgical resection at the Sun Yat-sen University Cancer Center between October 2007 and December 2009. All patients with LSCC were confirmed by pathology. The clinico-pathological features were collected and stored in our database. Patients were followed up every 3 months to assess survival status. Overall survival (OS) was recorded from the date of surgery to the date of death or last follow-up (31st May 2015), as previously reported [21]. This retrospective study on tumor material was approved by the Research Ethics Committee of Guangdong General Hospital & Guangdong Academy of Medical Sciences and Sun Yat-sen University Cancer Center (No.GDREC2018029H).

### Cell culture

Two human head and neck squamous carcinoma cell lines, Hep-2 and TU212, were purchased from the Cell Resource Center of Shanghai Institutes for Biological Sciences (Chinese Academy of Science, Shanghai, China). Cells were cultured in RMPI-1640 medium (Gibco, Waltham, MA,USA) supplemented with 10% fetal bovine serum (FBS; Gibco, Waltham, MA,USA), 100 U/mL penicillin, and 100 μg/mL streptomycin (Hyclone, Logan, UT, USA). Cells were cultured at 37 °C in 5%CO_2_/95% air in a humidified atmosphere. Both cell lines were routinely tested for mycoplasma contamination every 2 months in our lab. Tests for mycoplasma were negative for all duration of the experiment.

### Plasmid construction and cell transfection

Two lentivector-mediated short-hairpin OTX1 (sh-OTX1–1 and sh-OTX1–2) and non-targeting plasmids (sh-control) were designed and synthesized by GenePharma (Shanghai, China). A scramble, empty lentiviral vector was used as the control. Lipofectamine 2000 system was used for transfection. The lentivirial vectors carried puromycin resistance. The transfected cells were screened with 0.5 mg/ml of puromycin. Stably transfected cell lines were tested using quantitative reverse-transcriptase polymerase chain reaction (qRT-PCR) and western blot analysis. The transfection procedure was performed according to the manufacturer’s instruction. The following short-hairpin RNA (shRNA) sequences were used: sh-OTX1–1: GCAACACCTCGTGTATGCA; sh-OTX1–2: GCCGACTGCTTGGATTACA.

### Immunohistochemistry (IHC) analysis

LSCC tissues were paraffin-embedded and sliced. Slides were then subjected to de-paraffinization/rehydration, followed by the antigen retrieval in a microwave. Slides were blocked with 5% milk for 1 h at 25 °C, and then incubated with the corresponding anti-human OTX1 antibodies (1:400, MAD5602, Millipore, USA) at 4 °C overnight. After PBS washes, the slides were incubated with secondary antibodies at 25 °C for 1 h. Signals for each slide were developed in pre-made 0.05% diaminobenzidine (DAB) containing 0.01% hydrogen peroxidise (H_2_O_2_). The results of immunohistochemistry (IHC) were evaluated by two independent investigators who were blinded to the clinical and prognostic data based on the Shimizu criteria [22]. Based on the score criteria, the expression of OTX1 in LSCC tissues was graded as negative (−), weakly positive (+), moderately positive (++), and strongly positive (+++). The expression levels of OTX1 protein in LSCC tissues were dichotomized into the low (−/+) and high (++/+++) expression groups, respectively.

### Quantitative real-time PCR

Total RNA was extracted from LSCC tissue samples and cells using Trizol reagent (Invitrogen, USA) according to the manufacturer’s instructions. Complementary DNA (cDNA) was synthesized from total RNA using the cDNA Takara Kit (NHK, Japan). The transcriptional levels were detected in amplification by RT-PCR using the Power SYBR Green PCR Master Mix (NHK, Japan). Relative OTX1 mRNA expression was calculated using the 2^-ΔΔCT^ method and normalized to the internal control β-actin. The primers used in the study were as follows: OTX1 forward, 5′-GCGTCGTCGCTGAGTACAC-3′ and reverse, 5′-ACATGGGATAAGAGGCTGCTG-3′; β-actin forward, 5′-TCACCAACTGGGA CGACAT-3′ and reverse, 5′-GCACAGCCTGGATAGCAAC-3′.

### Western blot

Cells were lysed in ice-cold radioimmunoprecipitation assay (RIPA) lysis buffer containing 0.1% protease inhibitor. The concentration of total protein was measured using a bicinchoninic acid (BCA) assay kit (Boster, Wuhan, China). Equal amounts of protein were loaded onto 10% sodium dodecyl sulfate ployacrylamide gel electrophoresis (SDS-PAGE) and transferred to polyvinylidene fluoride (PVDF) membranes (Millipore, Bedford, MA, USA). After blocking with 5% skimmed milk, the membranes were incubated with anti-OTX1 (1:1000 dilutions, MAD5602, Millipore, MA, USA) and β-actin (1:3000 dilutions, CST4967, Cell Signaling Technology, Boston, USA) at 4 °C overnight. Subsequently, the membranes were washed with TBST buffer three times and incubated with the corresponding secondary antibodies for 1 h at room temperature. Finally, the immunoreactive bands on the membrane were visualized using enhanced chemiluminescence (ECL) reagents (Millipore, Plano, TX, USA).

### Cell proliferation

Cell proliferation was evaluated using the MTS (Qiagen, Hilden, German) assay. Briefly, Logarithmically growing cells were seeded in 96-well plates at a density of 3000 cells/well in triplicate. After incubation for 1, 2, 3, 4, 5, or 6 day, 20 μL of working solution containing MTS and serum-free RPMI-1640 medium was added into each well, followed by another incubation in humidified air with 5% CO_2_ at 37 °C for 2 h. The absorbance was measured at 490 nm using the Synergy™ Multi-Mode Microplate Reader (Biotek, Vermont, USA).

### Colony formation assay

For the colony formation assay, 500 cells were seeded in a 6-well plate and cultured in complete medium for 10 days. Cell colonies were fixed with methanol and stained with 0.2% crystal violet for 30 min at room temperature. The number of colonies was counted using Quantity One software (Bio-Rad, Hercules, CA, USA).

### Migration, and invasion assays

Cell migration was evaluated using the Transwell migration assay (8-μm pore; BD Biosciences). Briefly, cells were starved in serum-free medium for 2 h. Next, cells were resuspended in serum-free RPMI-1640 medium, and 200 μL of the cell suspension (3 × 10^6^ cells) was added to the upper chamber. A volume of 600 μL of RPMI-1640 medium containing 10% FBS was added to the bottom chamber. After 24 h, cells that attached to the lower surface of the membrane were fixed with methanol and stained with 0.2% crystal violet for 30 min. Finally, migratory cells were counted in five random fields. Th cell invasion assay was performed following the same procedures as described above, except that diluted Matrigel (Corning, New York, USA) was precoated on the upper well of the transwell chambers and incubated for 48 h.

### Xenografted tumors in nude mice

All animal experiments were performed in accordance with the guidelines for the Care and Use of Laboratory Animals of the National Institutes of Health. This study was approved by the Ethics Committee of Guangdong Provincial People’s Hospital (No.GDREC2018029A). Four-to-five-week old BALB/c nude mice were purchased from Beijing Vital River Laboratory Animal Technology Co., Ltd. and were raised under the specific pathogen-free environment at 20–22 °C and 40–60% humidity. After 1 week of adaptive feeding, the nude mice were randomly divided into six groups (*n* = 5 for each group) for Hep-2 and TU212 cells: Control, sh-OTX1–1, and sh-OTX1–2 respectively. To stably knock down OTX1, the shRNA-OTX1–1 and shRNA-OTX1–2 were constructed, packaged into lentiviruses, and used to infect Hep-2 and TU212 cells. After this, Hep-2 and TU212 cells infected with shRNA-OTX1–1 and shRNA-OTX1–2 or their corresponding controls were suspended in normal saline (1 × 10^6^ cells in 0.1 mL) and then subcutaneously injected into the right armpit of each mouse to establish a xenograft tumor mouse model. The tumor volume was calculated every 4 days. Twenty-eight days later, the mice were sacrificed under anesthesia. All animals were anesthetized by intravenous injection of barbiturate at a final concentration of 100 mg/kg. The tumors were isolated and fixed in 4% paraformaldehyde solutions and embedded in paraffin before being cut into 4 μm thick sections. The prepared sections were stained using haematoxylin and eosin (H&E) and Ki-67 following the routine staining procedure and examined using a microscope.

### Luciferase assay

MiR-129-5p or miR-129-5p-mut mimics were purchased from RiboBio Co., Ltd. (Guangzhou, China). DNA fragments from the 3′-untranslated region (UTR) of OTX1 containing the predicted complementary sites of miR-129-5p were constructed into a pGL3-basic plasmid (Addgene, Cambridge, USA). LSCC cells (1× 10^4^) were seeded in 48-well plates in triplicate and settled for 24 h. Next, the pGL3-OTX1–3′-UTR reporter plasmids (100 ng) plus 5 ng of pRL-TK Renilla plasmid (Promega, Madison, USA), and increasing amounts (10 and 50 nM) of negative control (NC) or miR-129-5p or miR-129-5p-mut mimic were co-transfected into LSCC cells using Lipofectamine LTX reagent (Invitrogen, Carlsbad, USA) according to the manufacturer’s recommendation. Luciferase and Renilla signals were measured 24 h after transfection using the Dual Luciferase Reporter Assay Kit (Promega, Madison, USA) according to a protocol provided by the manufacturer. The sequences of miR-129-5p and miR-129-5p-mut were 5′-cuuuuugcggucugggcuugc-3′ and 5′-cuuuuugcggucugguugugc-3′, respectively.

### Statistical analysis

All experimental assays were conducted at least three times. Data were represented as means ± standard deviation (SD). The two-tailed Student’s t-test was used to determine the statistical significance of differences between groups. Chi-square or Fisher’s exact test was used to analyse the categorical data. Survival analysis was performed using the Kaplan-Meier method and compared using the log-rank test. Statistical analysis was performed using the Statistical Program for Social Sciences 19.0 software (SPSS, CA, USA) and presented with GraphPad Prism 6.0 (GraphPad Software, CA, USA). Values of *P* < 0.05 were considered as statistically significant.

## Results

### OTX1 expression is correlated with the clinicopathological features and prognosis in patients with LSCC

**Fig. 1:**
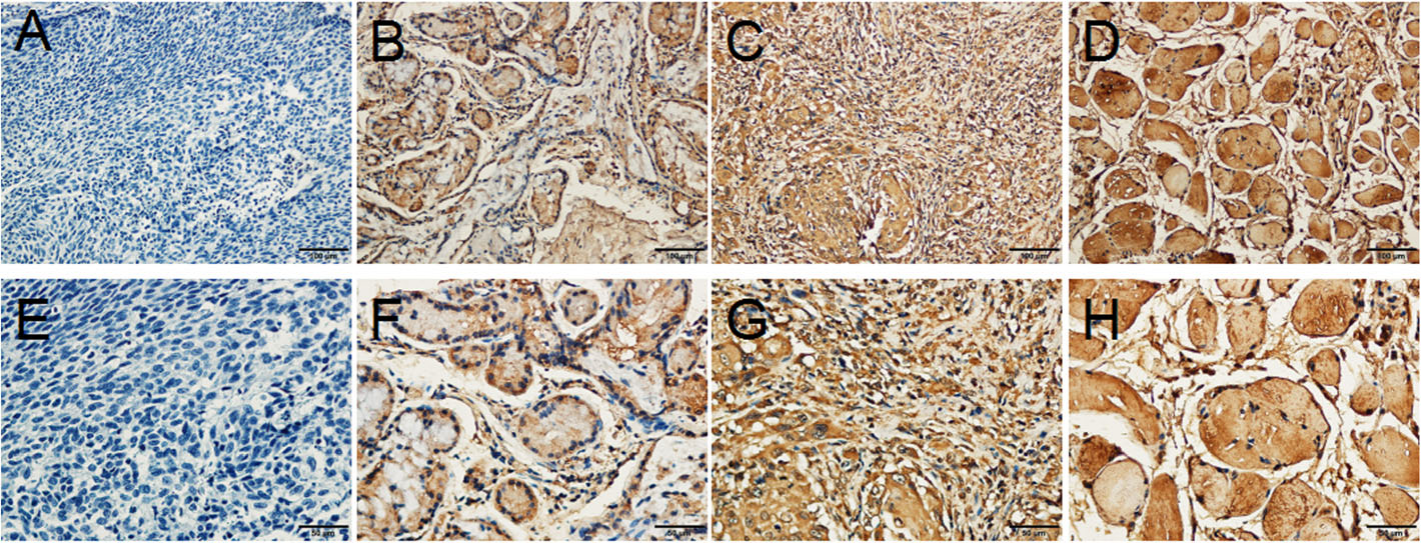
OTX1 expression in LSCC tissues. Immunohistochemical staining analysis was to performed for detection of OTX1 in human LSCC tissues. Representative images of negative (**a**, **e**), weakly positive (**b**, **f**), moderate positive (**c, g**), and strongly positive (**d**, **f**). Original magnification, × 100 (**a-d**); × 400 (**e-h**)

**Fig. 2:**
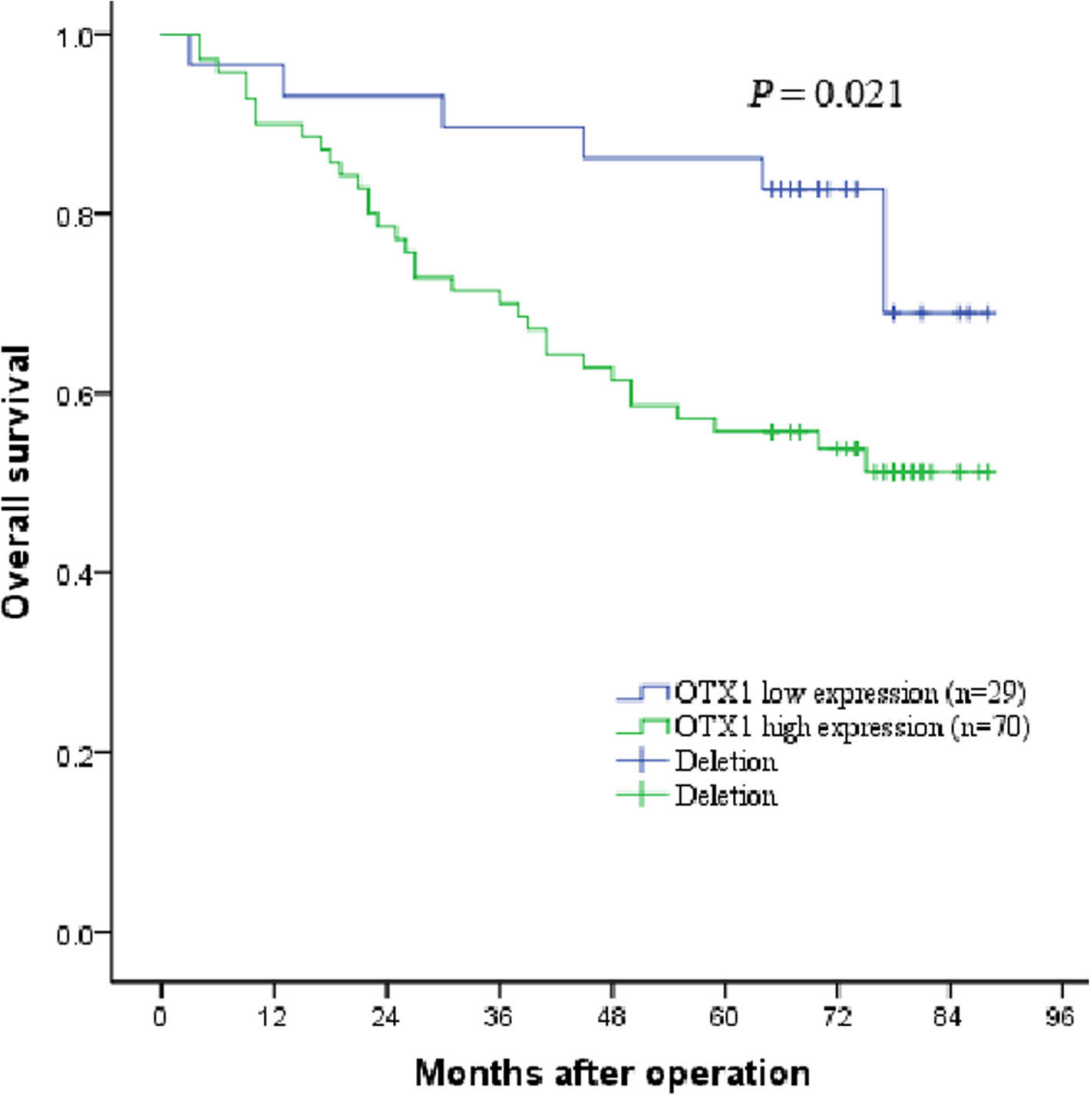
Association between OTX1 expression and prognosis in human LSCC. Kaplan-Meier survival analysis showed LSCC patients with OTX1 high expression had worse prognosis than those with OTX1 low expression

To investigate the clinical significance of OTX1 expression in patients with LSCC, we first examined the expression levels of OTX1 in human LSCC tissues using IHC (Fig. 1). The results revealed that high OTX1 expression was detected in 70.7% (70/99) of LSCC patient tissues. Next, we analyzed the correlation of OTX1 expression with clinicopathological features according to the IHC results. The expression level of OTX1 in LSCC was significantly correlated with lymph node metastasis and smoking history (all *P* < 0.05), while there were no significant correlations between the OTX1 expression and age, sex, alcohol consumption history, T classification, histological grade, or primary location in patients with LSCC (all *P* > 0.05) (Table 1). Lastly, we further found that the 1-, 3-, and 5-year overall survival (OS) rates were 90.0, 70.0, and 55.7% in LSCC patients with high OTX1 expression, respectively, compared to 96.6, 89.7, and 86.2% in LSCC patients with low OTX1 expression, respectively (*P* = 0.021, Fig. 2).

**Table 1:** Correlation between OTX1 expression and Clinicopathologic Variables of LSCC patients

		OTX1		
Variables	Cases (*n* = 99)	Low expression (*n* = 29)	High expression (*n* = 70)	*P*
Age (years)				
≤ 60	47	15 (66.9%)	32 (33.1%)	
> 60	52	14 (61.4%)	38 (38.6%)	0.586
Sex				
Male	95	27 (63.5%)	68 (36.5%)	
Female	4	2 (87.5%)	2 (12.5%)	0.353
Smoking history				
Yes	86	29 (64.0%)	57 (36.0%)	
No	13	0 (64.5%)	13 (35.5%)	0.013
Drinking history				
Yes	31	12 (62.5%)	19 (37.5%)	
No	68	17 (64.7%)	51 (35.3%)	0.165
T classification				
T1-T2	58	21 (69.0%)	37 (31.0%)	
T3-T4	41	8 (55.7%)	33 (44.3%)	0.072
Lymph node metastasis				
Negative	75	26 (65.5%)	49 (34.5%)	
Positive	24	3 (57.7%)	21 (42.3%)	0.038
Histological grade				
Well	39	14 (66.1%)	25 (33.9%)	
Moderately	44	11 (63.2%)	33 (36.8%)	
Poorly	16	4 (61.4%)	12 (38.6%)	0.508
Primary location				
Supraglottic	22	4 (65.4%)	18 (34.6%)	
Glottic	70	21 (55.3%)	49 (44.7%)	
Subglottic	7	4 (58.3%)	3 (41.7%)	0.139

*OTX1* Orthodenticle homeobox 1, *LSCC* Laryngeal squamous cell carcinoma

### Knockdown OTX1 expression suppresses the proliferation and colony formation of LSCC cells

**Fig. 3:**
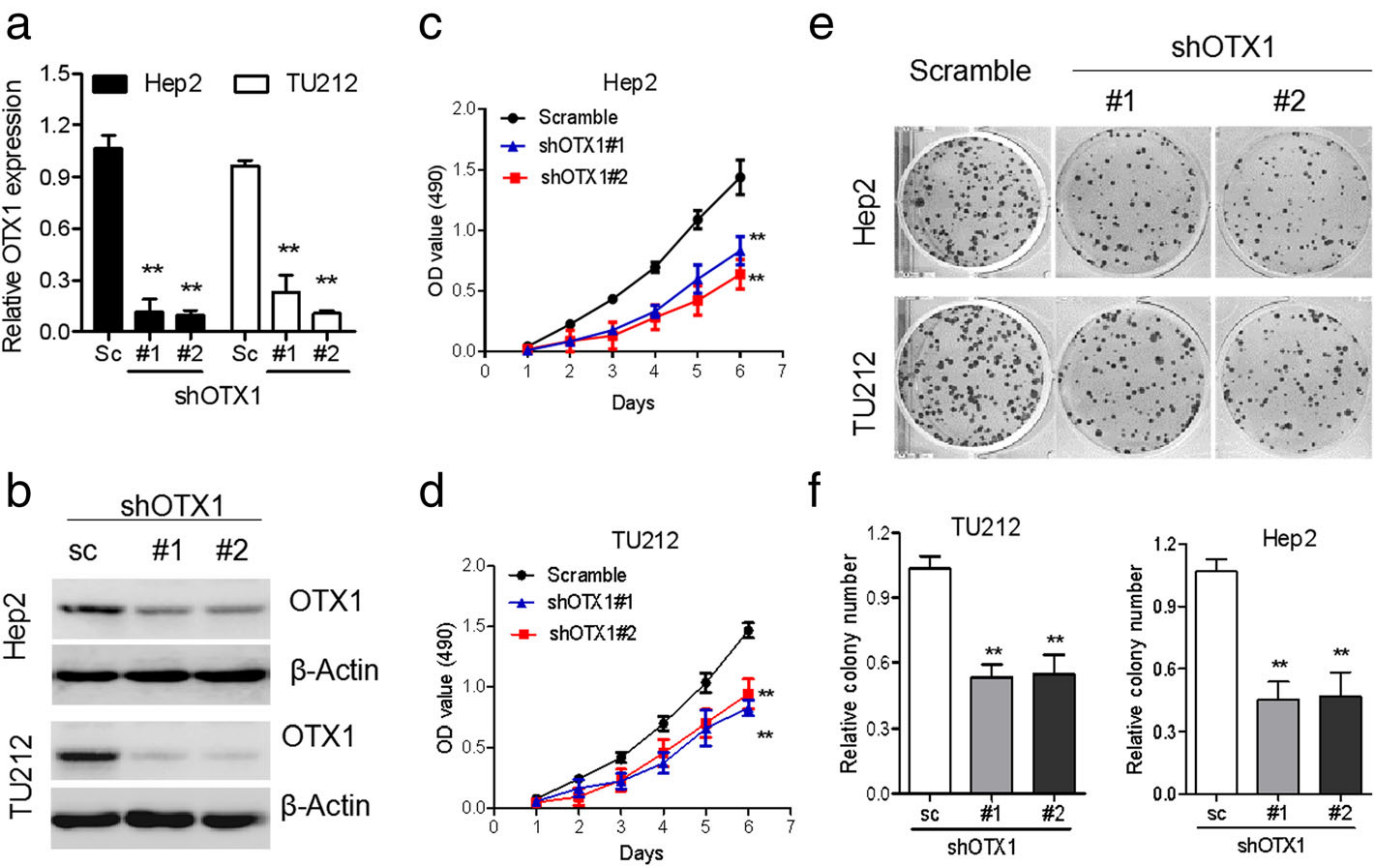
Knockdown OTX1 expression suppresses LSCC proliferation and colony formation in vitro. **a** OTX1 shRNAs inhibited the mRNA expression of OTX1 in Hep2 and TU212 cells using qRT-PCR. **b** OTX1 shRNAs inhibited the protein expression of OTX1 in Hep2 and TU212 cells using Western blot. **c** MTT assays showed that OTX1 downregulation inhibited the proliferation in Hep 2 cells. **d** MTT assays showed that OTX1 downregulation inhibited the proliferation in TU212 cells. **e** A colony formation assay demonstrated that OTX1 downregulation decreased the colony formation capacity of Hep2 and TU212 cells. **f** The quantitative graph of colony formation assay was performed to determine the colony formation capacity of Hep2 and TU212. SC: scrambled shRNA, #1: shOTX1–1, #2: shOTX1–2. *P < 0.05; **P < 0.01

To identify whether OTX1 plays a role in LSCC progression, we knocked down the expression of OTX1 by shRNAs and performed both cell proliferation and colony formation assays in LSCC cell lines Hep-2 and TU212. The inhibitory efficiency of shRNAs targeting OTX1 was evaluated based on qRT-PCR and western blot analysis. Compared to the scrambled control shRNA, shRNA-OTX1–1 and shRNA-OTX1–2 effectively reduced OTX1 expression in Hep-2 and TU212 cells (Fig. 3a and b). Next, we determined the effect of OTX1 expression on cell proliferation using the MTS assay. The results showed that OTX1 knockdown significantly inhibited the proliferation of Hep-2 and TU212 cells cells (Fig. 3c and d). Colony formation assays also indicated that OTX1 knockdown prominently suppressed colony formation ability of Hep-2 and TU212 cells (Fig. 3e and f).

### Knockdown OTX1 expression attenuates the migration and invasion of LSCC cells

**Fig. 4:**
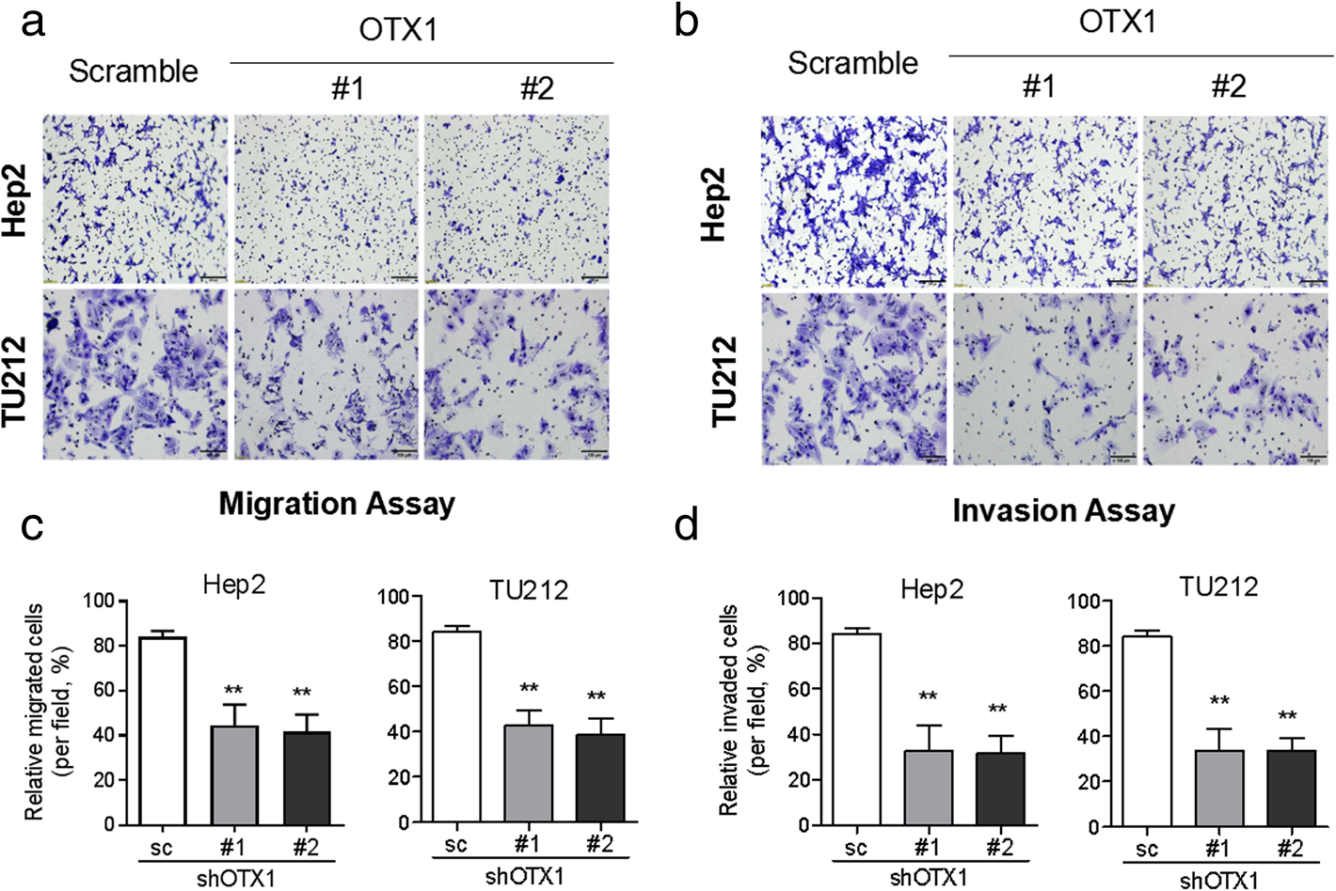
Knockdown OTX1 expression attenuates LSCC migration and invasion in vitro. **a** Transwell migration assay indicated that OTX1 downregulation suppresses the migration ability of Hep2 and TU212 cells. **b** Transwell invasion assay showed that OTX1 downregulation suppresses the invasion ability of Hep2 and TU212 cells. **c** The quantitative graph of Transwell migration assay. **d** The quantitative graph of Transwell invasion assay. SC: scrambled shRNA, #1: shOTX1–1, #2: shOTX1–2. *P < 0.05; **P < 0.01

To evaluate whether OTX1 affects the metastasis of LSCC cells, we analyzed the migration and invasion abilities of Hep-2 and TU212 cells after downregulating OTX1 expression. The migration abilities of Hep-2 and TU212 cells attenuated following knockdown of OTX1 compared with those in the matched control group based on the transwell migration assay (Fig. 4a and b). Compared to those in the matched control group, the invasion abilities of Hep-2 and TU212 cells attenuated after downregulating OTX1 expression based on the transwell invasion assay (Fig. 4c and d).

### Knockdown OTX1 expression inhibits tumor growth in vivo

**Fig. 5:**
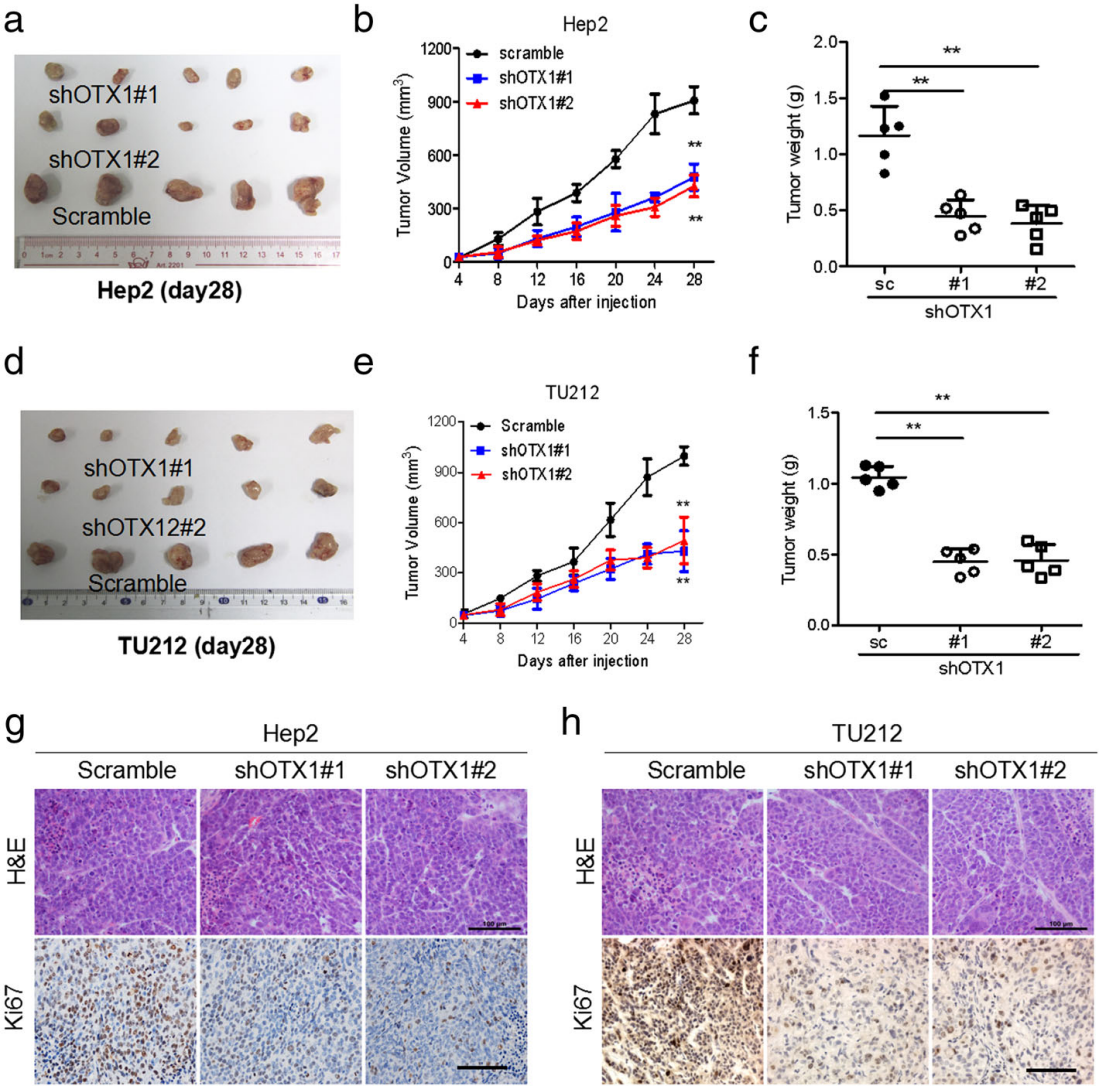
Knockdown of OTX1 inhibits tumor growth in a xenograft mouse model. **a** Representative images of day 28 tumors formed in nude mice that had been subcutaneously injected with Hep2 cells transfected with shOTX1–1, shOTX1–2 and SC. **b** The volumes of tumors dissected from the shOTX1–1 and shOTX1–2 group was lower than the SC group. **c** The tumor weight were significantly reduced following the injection of shOTX1–1 or shOTX1–2-expressing tumor cells compared with the SC group. **d** Representative images of day 28 tumors formed in nude mice that had been subcutaneously injected with TU212 cells transfected with shOTX1–1, sOTX1–2 and SC. **e** The volumes of tumors dissected from the shOTX1–1 and shOTX1–2 group was lower than the SC group. **f** The tumor weight were significantly reduced following the injection of shOTX1–1 or shOTX1–2-expressing tumor cells compared with the SC group. **g**, **h**. Representative HE and ki67 IHC staining image. SC: scrambled shRNA, #1: shOTX1–1, #2: shOTX1–2. **P < 0 .01

To further demonstrate the physiological relevance of OTX1 in LSCC in vivo, we investigated the effect of OTX1 on tumor growth using a xenotransplantation model. As expected, the OTX1 knockdown groups clearly showed reduced tumor growth and decreased tumor weight compared with those in the matched control groups (Fig. 5a-f). Moreover, OTX1 knockdown cells-induced tumor samples showed reduced proliferation indices (Ki-67 positivity) compared to those formed by matched controls in Hep-2 (Fig. 5g) and TU212 cells (Fig. 5h). In summary, these data suggested that knockdown of OTX1 inhibited tumor growth in vivo.

### OTX1 is negatively regulated by miR-129-5p

**Fig. 6:**
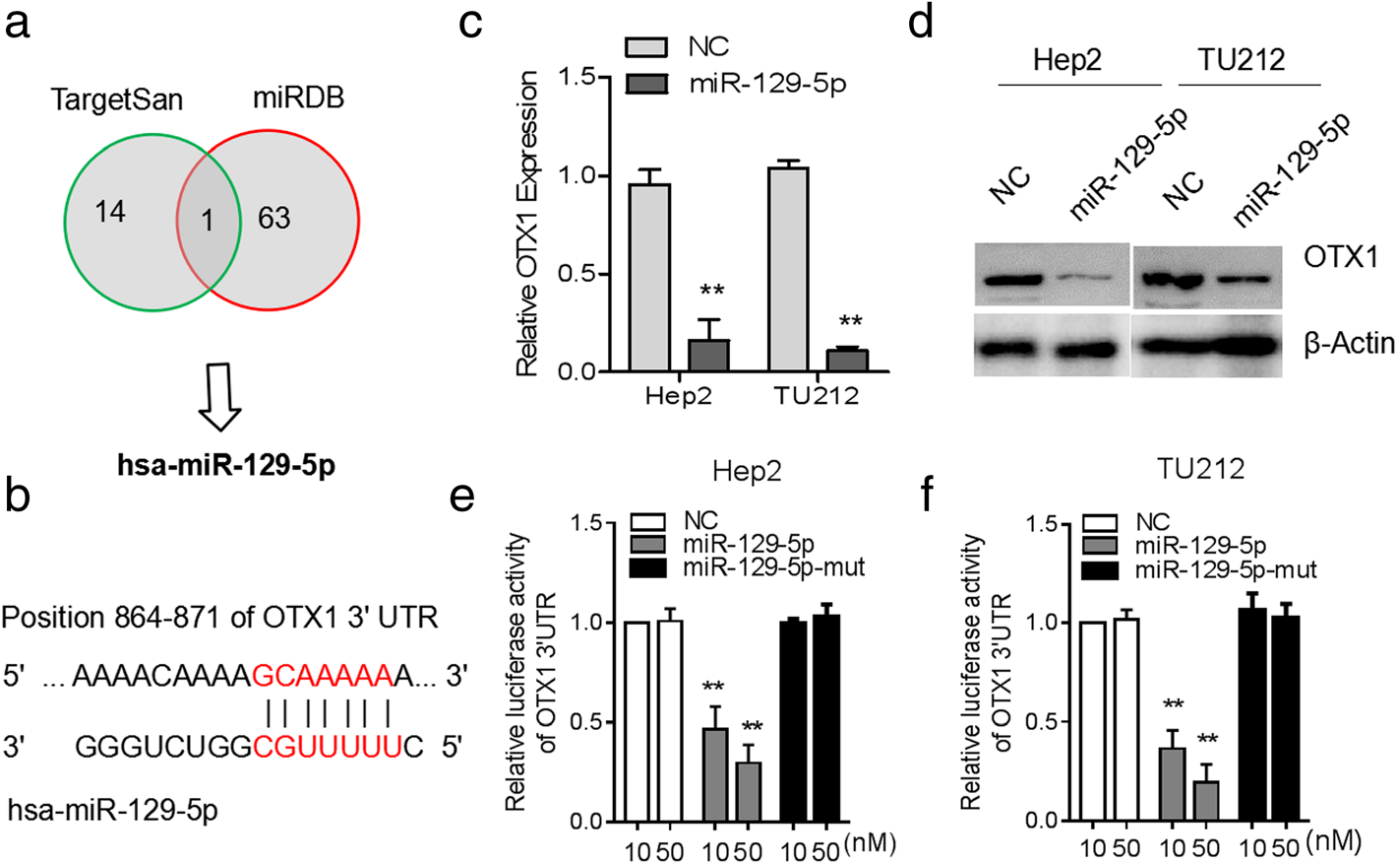
OTX1 acted as a direct target of the miR-129-5p in LSCC cells. **a** miR-129-5p could target OTX1 by using the human miRNA targets prediction tool miRanda and TargetSan. **b** Sequences of miR-129-5p and the potential binding sites in the 3’UTR of OTX1 is shown. **c** The expression of OTX1 was detected in Hep2 and TU212 cells using qRT-PCR after the transduction of miR-129-5p mimics. **d** The expression of OTX1 was detected in Hep2 and TU212 cells using Western blot after the transduction of miR-129-5p mimics. **e**, **f** Luciferase activity was determined in Hep2 and TU212 cells cotransfected with miRNAs (control mimics or miR-129-5p mimics) and a reporter vector containing miR-129-5p segments (WT or MUT) that bind to OTX1. **P < 0 .01

To further explore the mechanisms that regulate OTX1 expression in LSCC, we first predicted potential miRNAs that could bind to the 3′-UTR of OTX1 by Targetsan and miRDB. There were 14 and 63 miRNAs identified by Targetsan and miRDB, respectively. Among them, miR-129-5p simultaneously appeared in these two algorithms (Fig. 6a). Therefore, we chose miR-129-5p for further investigation. The predicted binding site of miR-129-5p to the sequence OTX1 was illustrated in Fig. 6b. At both the mRNA (Fig. 6c) and protein (Fig. 6d) levels, the expression of OTX1 was decreased after the overexpression of miR-129-5p by transfecting miR-129-5p into Hep2 and TU212 cells. Subsequently, we tested the direct binding affinity between miR-129-5p and the 3′-UTR of OTX1 using a luciferase reporter assay. The results indicated that the miR-129-5p mimics attenuated the luciferase activity of OTX1–3′-UTR, whereas the mutant miR-129-5p mimics did not suppress OTX1–3′-UTR luciferase activity (Fig. 6e and f). These results suggested that OTX1 might act as a direct target of miR-129-5p.

## Discussion

OTX1 is a homeobox gene that belongs to the OTX family (OTX1, OTX2, OTX3 and CRX). OTX1 plays a fundamentally important role in the development of the early human mammary gland and fetal retina [11, 14]. Recently, it has been reported that OTX1 is highly expressed in aggressive non-Hodgkin lymphoma, and medulloblastomas [20, 23]. High expression of OTX1 is also observed in solid tumors, such as gastric cancer, sinonasal carcinoma, and olfactory neuroblastoma [24, 25]. However, studies on the expression and role of OTX1 in human LSCC have not been reported.

In the current study, we first confirmed that OTX1 was overexpressed in LSCC tissues, and that high OTX1 expression in LSCC was associated with lymph node metastasis and poor prognosis. Next, we explored the potential role of OTX1 in tumor progression and metastasis. The knockdown of OTX1 expression by shRNA in human LSCC cells inhibited cell proliferation, migration and invasion in vitro and repressed tumor growth in vivo. We provide the first evidence that OTX1 plays a key role in the development and metastasis of LSCC. These results suggested that OTX1 might be a potential oncogene in laryngeal carcinogenesis.

Previous studies have shown that OTX1 is a target gene for p53 in breast cancer [19]. The expression of OTX1 is regulated by nitric oxide in the rat myenteric plexus after intestinal ischemia-reperfusion injury [26]. To clarify the mechanisms by which OTX1 is regulated in LSCC, we first predicted that OTX1 might interact with miR-129-5p via bioinformatics tools. We then discovered that the overexpression of miR-129-5p inhibited the expression of OTX1 at both the mRNA and proteins levels in LSCC cells. Furthermore, the luciferase reporter assay showed that miR-129-5p could directly bind to the 3′-UTR of OTX1. Aberrant expression of miR-129-5p has been observed in different types of cancers [27–29]. There have widely been reported that microRNAs (miRNAs) are strongly associated with the development and lymph node metastasis of LSCC [30, 31]. Our results suggested that miR-129-5p might target and suppress OTX1 in LSCC. Hence, the overexpression of miR-129-5p may be a potential therapeutic strategy for patients with LSCC with high OTX1 expression.

## Conclusions

In summary, our study demonstrated that OTX1 is highly expressed in LSCC and related with lymph node metastasis and poor prognosis. OTX1 promotes the progression and metastasis of LSCC in vitro and in vivo. MiR-129-5p is an upstream regulator of OTX1 expression, which might potentially shed light on new therapeutic method to alleviate LSCC progression.

## Data Availability

All the data generated and/or analyzed during this study are included in this published article (and its supplementary information files), and other datasets will be available from the corresponding author on reasonable request.

## Abbreviations

OTX1: Orthodenticle homeobox 1
LSCC: Laryngeal squamous cell carcinoma
OS: Overall survival
FBS: Fetal bovine serum
qRT-PCR: Quantitative real-time PCR
IHC: Immunohistochemistry
DAB: Diaminobenzidine
H2O2: Hydrogen peroxidise
SDS-PAGE: Sodium dodecyl sulfate ployacrylamide gel electrophoresis
PVDF: Polyvinylidene fluoride
ECL: Enhanced chemiluminescene
H&E: Haematoxylin and eosin
NC: Negative control
UTR: Untranslate region
SD: Standard deviation
SPSS: Statistical Program for Social Sciences
